# Inhibition of Dermatitis Development by Sopungsan in Nc/Nga Mice

**DOI:** 10.5487/TR.2008.24.1.017

**Published:** 2008-03-01

**Authors:** Yuba Raj Pokharel, Sung Chul Lim, Sang Chan Kim, Hoo Kyun Choi, Keon Wook Kang

**Affiliations:** 13 College of Pharmacy, Chosun University, Gwangju, 501-759 Korea; 23 Department of Pathology, College of Medicine, College of Medicine, Chosun University, Gwangju, 501-759 Korea; 33 Department of Prescription, College of Oriental Medicine, Daegu Haany University, Daegu, 706-060 Korea

**Keywords:** Atopic dermatitis, Anti-dermatitic activity, NF-κB, Sopungsan, TARC

## Abstract

Sopungsan (SS) is a traditional Korean decoction used for the treatment of dermatitis. The aim of this study is to confirm whether or not SS has a preventive effect on the development of atopic dermatitis in dinitrochlorobenzene-applied Nc/Nga mice. SS was administered orally to Nc/Nga mice, which led to the remarkable suppression of the development of dermatitis, as determined by a histological examination and the serum IgE levels. Moreover, SS inhibited the production of thymus- and activation-regulated chemokine (TARC) and its mRNA expression in a keratinocyte cell line, HaCaT, which had been stimulated with tumor necrosis factor-α (TNF-α) and interferon-γ (IFN-γ). Activation of the nuclear factor-κB (NF-κB) or activator protein-1 (AP-1) is one of key steps in the signaling pathways mediating induction of TARC. In this study, SS selectively suppressed NF-κB activation which may be essential for TARC expression in TNF-α/IFN-γ treated keratinocytes. The inhibitory effect of SS on NF-κB activation and TARC production might be associated with the anti-dermatitic effects of SS.

## INTRODUCTION

Atopic dermatitis is a common disease with an increasing incidence in industrialized countries. The clinical symptom of atopic dermatitis is characterized by pruritic skin lesions that are distinguished by infiltrating lymphocytes, macrophage and granulated mast cells (Hanfin and Rajka, 1980; Soter, 1989). Although topical steroids or anti-histamines are widely prescribed to manage this disease (Leung, 1997), long-term use of these agents causes serious side effects. Hence, a great deal of effort has been directed toward identifying safer compounds or herbal remedies that can inhibit the development of atopic dermatitis.

Some traditional Korean herbal prescriptions have been clinically applied as curative agents against atopic dermatitis. Sopungsan (SS) is one of herbal prescriptions used in Korea for the treatment of dermatitis. SS is a decoction consisting of 15 medicinal plants, *Spirodelae Herba*, *Glycyrrhizae Radix*, *Sophorae Radix*, *Angelicae Gigantis Radix*, *Cannabis Fructus*, *Akebiae Caulis*, *Ledebouriellae Radix*, *Rehmanniae Radix*, *Gypsum Fibrosum*, *Cicadae Periostracum*, *Forsythiae Fructus*, *Arcth Fructus*, *Atractylodis Rhizoma*, *Schizonepetae Herba* and *Sesami Semen Nigrum*. In Dongeuibogam, a classical oriental medicine literature SS is introduced as an effective prescription for skin diseases. Moreover, it has been recently reported that SS has inhibitory effect in contact dermatitis animal model (Park *et al.*, 2006).

Thymus- and activation-regulated chemokine (TARC/CCL17) is known as an endogenous ligand to CC chemokine receptor 4 (CCR4) and TARC binding to CCR4 results in attraction of Th2 cells and regulatory T cells (Imai *et al.*, 1997). TARC produced from basal keratinocytes plays a significant role in recruiting Th2-type lymphocytes to dermatitic skin lesions and a subsequent deterioration of dermatitis (Vestergaard *et al.*, 2000; Furukawa *et al.*, 2004).

In the present study, we first investigated whether SS administration protects the development of dermatitis in Nc/Nga mice topically applied with 1-chloro-2,4-dinitrobenzene (DNCB). We also determined the effect of SS on TARC production in a keratinocyte cell line, HaCaT to examine a possible mechanism for the anti-dermatitic effects of SS.

## MATERIALS AND METHODS

***Animals.*** The Institutional Animal Care and Utilization Committee of Chosun University approved all the animal procedures used in this study. Male Nc/Nga mice (25 g) were purchased from Joong-Ang Experimental Animals Co. (Seoul, Korea), placed in cages at a temperature between 20°C and 23°C with a 12 h light and dark cycle and a relative humidity of 50%. The animals were given commercial mouse chow (Purina, Korea) and water *ad libitum*. Controlled dermatitis was induced by topically applying 0.2% DNCB dissolved in acetone/olive oil (1 : 3) to the hair-removed back of the mice 3 times per week (Monday, Wednesday and Friday) for 10 weeks. The mice were then housed for 3 days without any further treatment. In case of the SS-treated group, SS was orally administered at a dose of 10 or 30 mg/mouse (6 times per week for 12 weeks) in their drinking water. Control animals received the vehicle. The mice were sacrificed on the day of the experiment (on 73 days after first applying the DNCB) and blood was collected from the *vena cava*. The skin tissues from the backs of the mice were excised and subjected to a histological examination.

***Preparation of sopungsan.*** All the medicinal plants were purchased from a herbal market (Daewon, Daegu, Korea) and was authenticated by Professor Sang Chan Kim, Daegu Haany University. Herbarium voucher specimens of SS were prepared and deposited in the herbarium of the Graduate School of Oriental Medicine, Daegu Haany University. The SS used in this study was composed of 12 g *Spirodelae Herba*, 4 g *Glycyrrhizae Radix*, 4 g *Sophorae Radix*, 4 g *Angelicae Gigantis Radix*, 4 g *Cannabis Fructus*, 4 g *Akebiae Caulis*, 4 g *Ledebouriellae Radix*, 4 g *Rehmanniae Radix*, 4 g *Gypsum Fibrosum*, 4 g *Cicadae Periostracum*, 4 g *Forsythiae Fructus*, 4 g *Arcth Fructus*, 4 g *Atractylodis Rhizoma*, 4 g *Schizonepetae Herba*, 4 g *Sesami Semen Nigrum*. A water extract of SS was prepared by boiling 1,360 g SS in 4 l of distilled water at 100°C for 3 h. The extract was centrifuged 3,000 g for 3 min and the resulting supernatant was lyophilized to produce a powder (184.7 g), which was stored at 4°C.

***Other materials.*** The recombinant TNF-α and IFN-γ were obtained from Peprotech Inc. (Rocky Hill, NJ). The human TARC and mouse IgE enzyme linked-immunosorbent assay kits were purchased from R&D Systems (Minneapolis, MN) and Shibayagi Co. (Gunma, Japan), respectively. 1-chloro-2,4-dinitrobenzene (DNCB) was obtained from Aldrich (Milwaukee, WI). Most of the reagents used for the molecular studies were purchased from Sigma (St. Louis, MO).

***Cell culture.*** The HaCaT cells, a human keratinocyte cell line were cultured in Dulbecco's modified Eagle's media (DMEM) supplemented with 10% fetal bovine serum (FBS), 100 mg/ml penicillin and 100 mg/ml streptomycin, in 5% $CO_2$ at 37°C. The production of TARC was stimulated by incubating HaCaT cells with 20 ng/ml TNF-α and 20 ng/ml IFN-γ. The SS extract was dissolved in sterile PBS and added to the incubation medium 10 min before adding TNF-α/IFN-γ.

***Enzyme linked immunosorbent assay (ELISA).*** Both the serum levels of IgE in the Nc/Nga mice and the supernatant levels of TARC in the HaCaT cells were measured according to the manufacturer's instructions. The absorbance was measured at a wavelength of 450 nm, and the concentration was calculated from a standard curve using serially diluted IgE or recombinant TARC.

***Histopathologic examination.*** The skin tissues were isolated from each mouse and fixed in 10% formalin in 50 mM of a phosphate buffer (pH 7.0) for 24 h at 4°C. The skin tissues were subsequently embedded in paraffin, sectioned (4 μm), stained with hematoxylin and eosin, and examined by optical microscopy (Olympus, Tokyo, Japan). A certified pathologist analyzed and scored the samples in a blinded manner. A minimum of 2 sections per experimental animal were examined for the presence and degree of the following: incrustation, thickening of the epidermis, epidermal necrosis, bleeding, hyperkeratosis, and inflammation of the epidermis and dermis.

***Reverse transcription-polymerase chain reaction (RT-PCR).*** The total RNA was isolated using the guanidium-isothiocyanate procedure, as described elsewhere (Lee *et al.*, 2005). The total RNA (1.0 mg) obtained from the cells was reverse-transcribed using an oligo(dT) 20mer as a primer and M-MLV reverse transcriptase (Bioneer, Eumsung, Korea) to produce the cDNAs. PCR was performed using the selective primers for the human TARC (sense primer: 5'-ACTGCTCCAGGGATGCCATCGTTTTT-3', antisense primer: 5'-ACAAGGGGATGGGATCTCCCTCACTG-3') (270 bp) and the S16 ribosomal protein (S16r) genes (sense: 5'-TCCAAGGGTCCGCTGCAGTC-3', antisense: 5'-CGTTCACCTTGATGAGCCCATT-3') (152 bp). PCR was carried out for 35 cycles using the following conditions: denaturation at 98°C for 10 sec, annealing at 60°C for

0.5 min, and elongation at 72°C for 1 min. The band intensities of the amplified DNAs were compared after visualization on a UV transilluminator.

***Transient transfection and NF-κB and AP-1 reporter gene assay.*** The cells were plated on a 12-well dish and transfected the following day. The promoter activity was determined using a dual-luciferase reporter assay system (Promega, Madison, WI). Briefly, the cells were transiently transfected with 1 μg of pNF-κB-Luciferase or pAP-1-Luciferase plasmid and 20 ng of the pRL-SV plasmid (*Renilla* luciferase expression for normalization) (Promega, Madison, WI) using the Genejuice® reagent (Novagen, Madison, WI), and then exposed to TNF-α (20 ng/ml)/IFN-γ (20 ng/ml) for 18 h. The firefly and *Renilla* luciferase activities in the cell lysates were measured using a luminometer (Turner Biosystems; TD-20, Sunnyvale, CA). The relative luciferase activity was calculated by normalizing the NF-κB or AP-1 promoter-driven firefly luciferase activity to that of *Renilla* luciferase.

***Statistics.*** The differences between the treatment groups were examined using unpaired Student's *t*-test. The criterion for statistical significance was set at either $p < 0.05$ or $p < 0.01$.

## RESULTS

***SS protects progression of dermatitis in Nc/Nga mice topically applied with DNCB.*** Nc/Nga mice develop dermatitis with depilation and hemorrhage when housed in conventional conditions but not in specific pathogen-free conditions (Matsuda *et al.*, 1997). However, the Nc/Nga mice bred in conventional conditions for 10 weeks showed only mild dermatitis and even higher variations in our experimental condition. Hence, 250 μl of 0.1% DNCB was topically applied to the back of the mice for 10 weeks (3 times a week) in order to induce strong and controlled dermatitis. The DNCB applied Nc/Nga mice showed progressive diffuse erythematous changes, scaling, lichenified areas, oozing and crusting on the back (Fig. 1A) as well as histological findings of hyperkeratosis, parakeratosis, acanthosis with varying degrees of spongiosis, exocytosis of mononuclear cells in the epidermis and the infiltration of inflammatory cells into the upper dermis (Fig. 1A), which suggests that the chronic application of DNCB can accelerate the development of dermatitis in Nc/Nga mice.

Next, we examined whether SS administration prevents DNCB-induced dermatitis in Nc/Nga mice. SS (10

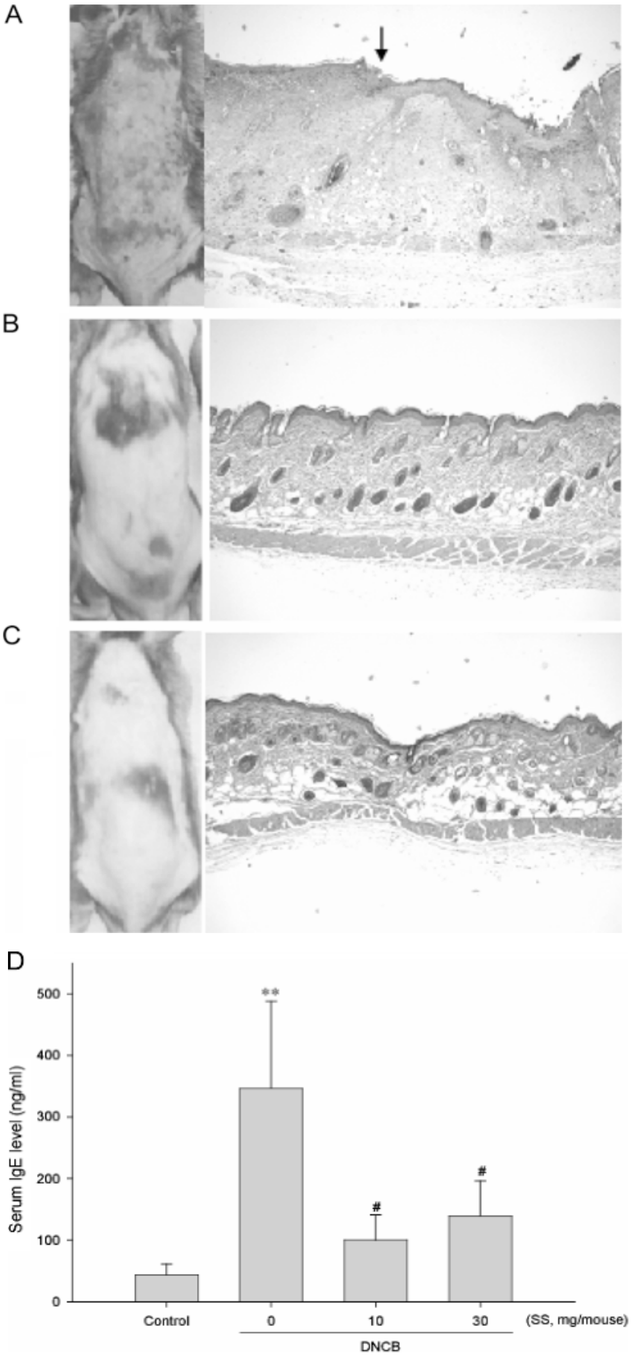

**Fig. 1.** Effects of SS on the development of dermatitis and the serum IgE levels in DNCB-applied Nc/Nga mice. Representative photographs (Left panel) and hematoxylin/eosin staining (Right panel, × 100) of skin sections from Nc/Nga mice treated with DNCB (0.1%, 3 times per week for 10 weeks) (A), DNCB + SS (10 mg/mouse, 6 times per week for 10 weeks) (B) and DNCB + SS (30 mg/mouse, 6 times per week for 10 weeks) (C). (D) The serum concentrations of IgE were determined using mouse IgE-specific ELISA kits. The results shown represent the means ± SEM of 5~7 different samples (significant compared with control, $^{**}p < 0.01$; significant compared with the DNCB-applied Nc/Nga group, $^{\#}p < 0.05$).

**Table 1.** Effect of SS on pathological parameters of dermatitis in Nc/Nga mice

| Group \ Parameter | IncruR | E/Thick | E/Nec | Inf/Epi |
|---|---|---|---|---|
| Nc/Nga (Vehicle) | 0.03 ± 0.03 | 0.10 ± 0.01 | 0 | 0 |
| Nc/Nga (DNCB) | 0.31 ± 0.05 | 0.17 ± 0.01 | 2.5 ± 0.5 | 2.8 ± 0.2 |
| Nc/Nga (DNCB) + SS 10 mg/mouse | 0 | 0.09 ± 0.001 | 0 | 0 |
| Nc/Nga (DNCB) + SS 30 mg/mouse | 0 | 0.08 ± 0.005 | 0 | 0 |

IncruR: incrustation ratio = Total length of incrusted areas/Total length of each sample.
E/Thick: epidermal thickness.
E/Nec: epidermal necrosis (no necrosis: 0, mild: 1, moderate: 2, severe: 3).
Inf/Epi: inflammatory cells infiltration in the epidermis (no infiltration: 0, mild: 1, moderate: 2, severe: 3).
Data represent the means ± SEM of 3~6 different samples.

and 30 mg/mice) decreased the intensity of dermatitis in the DNCB-applied Nc/Nga mice. Histological studies revealed that thickening of the epidermis and infiltration of inflammatory cells were significantly lower in the SS-treated groups [Fig. 1B (SS 10 mg/mice), 1C (SS 30 mg/mice) and Table 1]. Multiple analyses of the histology samples confirmed that SS significantly reduced the indices of dermatitis compared with that induced by DNCB alone (i.e. Epidermal thickness: 0.17 (DNCB alone) *vs.* 0.09 (SS 10 mg/mouse) or 0.08 (SS 30 mg/mouse) (Table 1).

***SS decreases serum IgE increase in Nc/Nga mice topically applied with DNCB.*** In Nc/Nga mice, high levels of serum IgE are detected and the IgE increase is believed as one of indices for the development of dermatitis (Matsuda *et al.*, 1997; Kotani *et al.*, 2000). We found that chronic application of DNCB for 10 weeks significantly enhanced the serum IgE levels compared with the vehicle-treated group and the increase in serum IgE levels were reversed in the SS-treated groups (Fig. 1D). In the mice treated with 10 and 30 mg SS, the inhibition percentage of serum IgE was 71 and 60%, respectively (Fig. 1D). These results demonstrated that the oral administration of SS prevents the development of dermatitis in Nc/Nga mice.

***SS inhibits TARC production in HaCaT cells treated with TNF-α/IFN-γ.*** TARC is a chemokine secreted from dermal dendritic cells and is involved in the development of Th2-mediated inflammation such as atopic dermatitis (Imai *et al.*, 1997; Sandoval-Lopez and Teran, 2001). Vestergaard *et al.* (2000) also showed that TARC actively participate in the pathogenesis of atopic dermatitis in Nc/Nga mice. In cell culture studies using primary human keratinocytes or HaCaT cells, TNF-α and IFN-γ synergistically induce TARC production, which might play a role in the development of atopic dermatitis (Vestergaard *et al.*, 2000). Because SS showed suppressive effect on the development of dermatitis in the DNCB-applied Nc/Nga mice, we hypothesized that SS could affect the production of TARC in HaCaT cells by TNF-α/IFN-γ.

In ELISA assay using a human TARC specific antibody, we found that the level of TARC production was

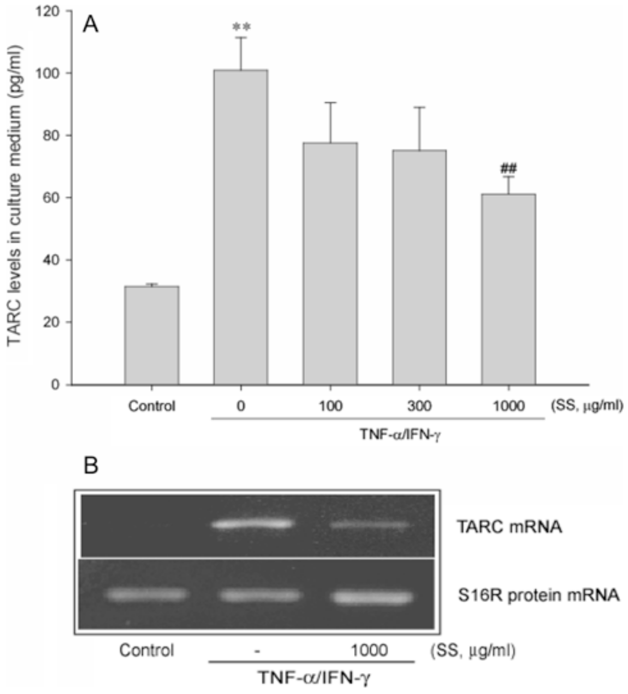

**Fig. 2.** Effects of SS on the TNF-α/IFN-γ-induced production of TARC. The HaCaT cells were incubated in a medium containing SS for 10 min and treated with TNF-α/IFN-γ (20 ng/ml, each) for 24 h (protein) or 3 h (mRNA). (A) Effect of SS on the TNF-α/IFN-γ-induced production of TARC in HaCaT cells. The concentration of TARC in the medium was determined using TARC-specific ELISA assays. The results shown represent the mean ± SEM of 4 different samples (significant compared with control, **$p < 0.01$; significant compared with TNF-α/IFN-γ-treated group, ##$p < 0.01$). (B) The TARC mRNA expression levels were determined by RT-PCR analysis. The mRNA expression of the S16 ribosomal protein was comparable among the samples.

increased in cells treated with TNF-α (20 ng/ml)/IFN-γ (20 ng/ml) for 24 h and the increase in TARC production was significantly inhibited by 1000 μg/ml SS pretreatment (Fig. 2A). To further determine whether SS inhibits the transcription of the TARC gene, the expression levels of TARC mRNA in HaCaT cells exposed to TNF-α (20 ng/ml)/IFN-γ (20 ng/ml) for 3 h were measured. The expression of TARC mRNA was increased markedly by TNF-α/IFN-γ, and this increase was diminished in HaCaT cells pretreated with 1000 μg/ml SS (Fig. 2B). In contrast, mRNA levels of S16 ribosomal protein were similar in all samples (Fig. 2B). Hence, SS may target transcription process of TARC gene.

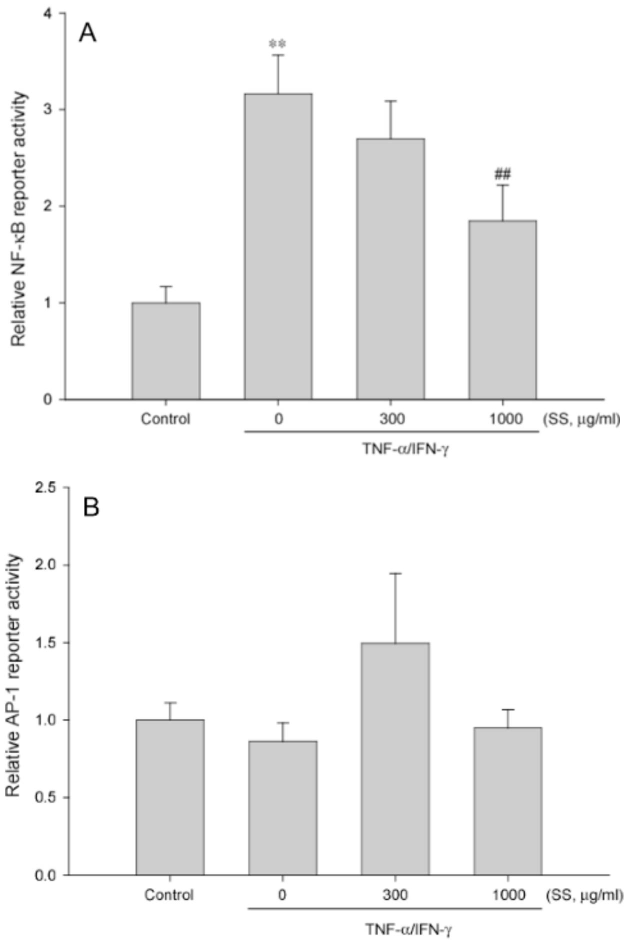

**Fig. 3.** Effects of SS on the NF-κB and AP-1 activities. (A) NF-κB reporter gene analysis. The induction of luciferase activity by TNF-α/IFN-γ in HaCaT cells transiently transfected with the pNF-κB-Luc construct, which contained the three-times repeated NF-κB binding sequences, was confirmed using a luminometer. A dual luciferase reporter gene assay was performed on the lysed cells that had been co-transfected with the pNF-κB-Luc (firefly luciferase) and pRL-SV (*Renilla* luciferase) (a ratio of 100 : 1) after exposing them to TNF-α/IFN-γ (20 ng/ml, each) and SS (300 or 1000 μg/ml) for 18 h. The activation of the reporter gene was calculated as the relative change in the *Renilla* luciferase activity. The data represent the mean ± SD of 4 separate experiments (significant compared with control, $^{**}p < 0.01$; significant compared with the TNF-α/IFN-γ-treated group, $^{\#\#}p < 0.01$). (B) AP-1 reporter gene analysis. The cells were transfected with the pAP-1-Luc plasmid, and reporter gene analysis was performed as reported in panel (A). The data are represented as a mean ± SEM of 4 separate experiments.

***SS inhibits NF-κB activation in HaCaT cells stimulated with TNF-α/IFN-γ.*** The promoter region of the TARC gene contains putative NF-κB and AP-1 binding sites (Nakayama *et al.*, 2004) and recent studies also showed that the transcriptional regulation of TARC gene is also controlled by the NF-κB or AP-1 activity (Berin *et al.*, 2001; Komine *et al.*, 2005; Nakayama *et al.*, 2004). Thus, we performed reporter gene analyses using luciferase reporter plasmids containing the NF-κB or AP-1 binding sequences to determine if the suppressive effect of SS on the induction of TARC gene occurs alongside the inhibition of NF-κB or AP-1. TNF-α/IFN-γ (20 ng/ml each, 18 h) caused a 3.2-fold increase in NF-κB reporter activity (Fig. 3A), which was inhibited by pretreating the cells with 1000 μg/ml of SS for 10 min (Fig. 3A). However, TNF-α/IFN-γ did not increase the AP-1 reporter activity in HaCaT cells. Moreover, the basal AP-1 reporter activity was not affected by 300 or 1000 μg/ml SS (Fig. 3B). These results show that SS selectively inhibits the NF-κB activation process, and is associated with its suppressive effect on TARC induction caused by inflammatory insults.

## DISCUSSION

Here, we found that topical application of DNCB on the back of Nc/Nga mice was an efficient tool to induce severe and controlled dermatitis within 10 weeks. In contrast, DNCB itself did not cause any pathological changes in ICR mice (data not shown), which suggests that the dermatitis observed in our system is mainly the result of the DNCB-induced potentiation of spontaneous dermatitis in Nc/Nga mice. Chronic administration of SS effectively prevented the development of dermatitis, as evidenced by histological examination and serum IgE determination. SS is often prescribed for patients with atopic dermatitis in Korean oriental medicine hospitals, and our results provide a scientific evidence to support its clinical efficacy.

TARC functions as a selective chemoattractant and assists in the recruitment and migration of Th2 cells expressing CCR4 (Sandoval-Lopez and Teran, 2001). In fact, TARC antibody therapy reduces the development of allergic airway inflammation and hyper-responsiveness (Kawasaki *et al.*, 2001). Hence, TARC has been suggested to be an important mediator that exaggerates atopic dermatitis. The clinical and pharmacological efficacy of SS against dermatitis might partly be associated with its inhibitory actions on TARC production in keratinocytes because both the release of TARC and its mRNA expression were diminished in keratinocyte cell line exposed to SS. The promoter of the human TARC gene contains several homologous consensus sequences for the binding of transcription factors including STAT, NF-κB and AP-1 (Nakayama *et al.*, 2004). Among these transcription factors, it was reported that either NF-κB or AP-1 is important for the transcription of the human TARC gene (Komine *et al.*, 2005; Nakayama *et al.*, 2004). In the present study, we showed that SS selectively inhibited TNF-α/IFN-γ-inducible NF-κB reporter activity. Therefore, the inhibition of NF-κB by SS may be related with the suppression of TARC induction in TNF-α/IFN-γ-treated keratinocytes.

In summary, the chronic administration of SS, at doses of 10 or 30 mg/mice/day prevents the development of dermatitis in Nc/Nga mice. These beneficial effects may at least in part be associated with the inhibitory effect of SS on NF-κB-mediated TARC production in keratinocyte in the dermatitic lesions.

## ACKNOWLEDGEMENT

This study was supported by the Oriental Medicine R&D Project, Ministry of Health & Welfare, Republic of Korea (Grant No. B050035).
